# Role Participation: A Comparison across Age Groups in a Norwegian General Population Sample

**DOI:** 10.1155/2018/8680915

**Published:** 2018-05-10

**Authors:** Tore Bonsaksen

**Affiliations:** ^1^Department of Occupational Therapy, Prosthetics and Orthotics, Faculty of Health Sciences, OsloMet - Oslo Metropolitan University, Oslo, Norway; ^2^Faculty of Health Studies, VID Specialized University, Sandnes, Norway

## Abstract

**Background:**

Life events, illness, and disability may alter a person's role participation, in which case occupational therapy may be an appropriate intervention. However, role participation data derived from the general population, which is required for meaningful comparison, is largely missing. This study is aimed at describing past, present, and anticipated role participation in a general population sample from Norway and at examining differences in current role participation between age groups.

**Methods:**

In 2015, a sample of 140 persons (age range 19–94 years, 65% females) from the Norwegian general population completed the Role Checklist at one occasion. The data were analyzed descriptively and with chi-square tests and one-way analysis of variance.

**Results:**

The most frequent role was a home maintainer (93.6%), and the least frequent was a religious participant (7.1%). Participants aged 65 years and above had fewer roles compared with their younger counterparts and had to a larger extent experienced role loss over the course of their lives.

**Conclusions:**

Role continuity was the most prevalent role pattern in the total sample, whereas role loss appeared to be the most prevalent role pattern among those in the oldest age group. Rehabilitation services in general and participation-focused occupational therapy in particular may profit from assessing role participation in clients and potentially target roles through intervention.

## 1. Introduction

A core aim for occupational therapy services is to promote participation in society by enabling the performance of meaningful, everyday-life occupation [1, 2]. Recently, researchers have suggested that participation may be understood primarily as participation within social roles [3–5]. This idea links the participation concept of the International Classification of Functioning, Disability and Health (ICF) [6] directly with the Model of Human Occupation (MOHO) theory [1], in which social roles are viewed as the main organizing principle of the person's occupational behavior. By performing the tasks and chores associated with social roles, one participates in society. Acting within social roles is *inter*acting—acting in relationship with others, directly or indirectly. The role content is constituted from the ongoing interaction between the person holding the role and their social environment. In other words, acting according to role expectations within the social context constitutes the theoretical definition of role participation [1].

Roles are partly prescribed—for example, one is born as someone's son or daughter. Over the life course, other social roles are acquired, as one takes on various social positions in relationship to others, for example, the role of a student or a teacher. The roles we enact at a given point in time influence what we do and how we do it. With practice, the person gradually identifies with their roles such that their role behaviors come naturally and without much consideration. At this point, the role is internalized within the person—it has become a part of the person's identity [1]. However, when reflecting on the value of roles, a study of Norwegian occupational therapy students showed that most of the performed roles, as reported on the employed Role Checklist, were also highly valued [7]. Subjective well-being among university students in Jordan was associated with having more roles and perceiving roles as more valuable [8], and similarly, participating in more valued roles was associated with higher quality of life among persons who had undergone liver transplantation [9].

Adverse life events, illness, and disability may limit the person's performance in social roles and may consequently change or restrict their social participation [1]. Several studies have been in support of this reasoning. For example, one study found more problems in academic, leisure, and work roles in adolescents with mental illness compared to healthy peers [10]. One year after the landfall of Hurricane Ike at the coast of Texas in September 2008, students in the area experienced that some previous roles were retained whereas others had been disrupted [11]. Scott's [9] study of liver transplant recipients also showed a pattern of disengagement with previously enacted roles, whereas Dickerson and Oakley's [12] study found a consistent pattern of more current roles among nondisabled community-living individuals compared with individuals who had physical or psychosocial disabilities.

However, not only do roles change as an effect of specific events but they also change over the natural life course, and occupational therapy may be relevant for remediating problematic role transitions [13]. In the words of Lee and Kielhofner [14], “Society expects and structures role transitions at various life stages such as entering and exiting the student role, beginning work, and retiring. People also choose to enter and leave roles” (p. 67). One perspective of role patterns across time, encompassing change as well as stability, was provided in a Canadian study of adults with severe mental health problems [15]. Using the Role Checklist [16] to examine roles in a time perspective, the researchers conceptualized role stability in terms of continuity (having the role in the past, present, and foreseen future) and absence (not having the role in the past, nor in the present or foreseen future). Role loss was conceptualized as having the role in the past, but not at present. Conversely, role gain was conceptualized as not having the role in the past, but having it at present. The concept “role change” was used to describe a situation where a role was not enacted at present but was expected in the future.

Conceptually, though, the above example of role change is more precisely one of the “expected role gains,” which is merely one of several ways roles can change over time. Adding to this idea, we may therefore differentiate between two aspects of role change: expected role gain and expected role loss. A person heading towards retirement from work could exemplify how role change may involve both aspects. Retirement implies losing the role of a worker (role loss) but may also open up the possibility of taking up, for example, the role of a choir member or a volunteer for a charity organization (role gains).

Two contrasting theories of “successful aging” have traditionally been put forward: the activity theory and the disengagement theory [17, 18]. The latter view emphasizes accepting losses (e.g., of functional ability, activities, roles, and friends) and disengagement from active life, while the former emphasizes holding on—as far as possible—to the roles and activities of middle age. While both views seem valid, recent research evidence seems to favor the activity theory, essentially stating that having a good life in older years is not about giving in, but holding on. For example, Menec's [19] six-year longitudinal study of elderly persons demonstrated that in general, social and productive activities were positively related to higher levels of happiness, better functioning, and lower mortality. As activities are thought to be organized mainly according to roles [1], one could also suggest that having more roles might indicate greater satisfaction with life. Research has shown that currently held roles were often valued roles among students [7] and that participation in more valued roles was related to greater quality of life among liver transplant recipients [9]. However, the notion that having more roles is associated with greater life satisfaction, regardless of the person's valuation of these roles, is an assumption in need of further inquiry.

In summary, the available literature suggests that participating in more valued roles is a source of contentment and satisfaction with life. Moreover, it suggests that adverse life events, illness, and disability can potentially disrupt the individual's social roles. However, role participation is also affected by the normal progression through life. Thus, aging may often imply a need for adapting to new circumstances. Such adaptation may be a difficult process, and occupational therapy may be appropriate as a means to remediate disrupted participation in roles. However, existing studies have largely employed samples with highly specific characteristics, frequently related to a specific illness or disability (see, e.g., [9, 20, 21]). The one study providing comparisons with a general population sample [12] is both dated and concerned with the US context only. To be able to interpret role participation data from different clinical groups, one should preferably be able to compare with current data derived from the general population living in the relevant cultural context.

The present study is aimed at describing past, present, and anticipated role participation in a general population sample from Norway. Second, differences in current role participation between individuals in different age groups were examined. Lastly, building on the procedure described by Hachey and coworkers [15], we described the participants' role patterns.

## 2. Methods

The study employed a cross-sectional design, utilizing data from an assessment at one point in time. It serves as a Norway-specific substudy associated with a larger cross-cultural study of roles and their links with MOHO as a theoretical framework [3]. The data for the study was collected early in 2015.

### 2.1. Sample and Recruitment

The sample was a convenience sample of persons from the general population, recruited by personal contact made by nine occupational therapy students and occupational therapist colleagues in Oslo and in the surrounding metropolitan area. Age under 18 years was the only exclusion criterion. The participants were recruited with the aim of making the sample as diverse as possible, representing a blend of men and women and people in different age groups. The participants knew the recruiter in person, or they knew someone known to the researcher, for example, in cases where they were that person's friend or spouse (“snowballing recruitment”). To ensure a diverse sample composition, the recruiters were asked to include both men and women and to continue to include participants until the sample consisted of at least 20 persons in each of the predefined age groups. The age groups represented young persons (aged 18–24), adults (aged 25–44), middle-aged persons (aged 45–64), and elderly persons (aged 65 years and above). Unfortunately, however, the recruitment of participants to the oldest age group was not fully completed due to time restrictions on the part of the recruiters. On the other hand, more than 20 participants were recruited in the other age groups, increasing the statistical power of the analyses.

### 2.2. Measurement

Role participation was assessed with the Role Checklist [16]. The instrument is widely used—it is available in 13 languages and remains one of the most commonly used assessments in American occupational therapy practice [22]. It is a short self-report assessment that captures a person's perception of their participation in 10 defined roles. The roles are student, worker, volunteer, home maintainer, caregiver, friend, family member, hobbyist/amateur, religious participant, and participant in organizations. Part one of the Role Checklist, which was the only part employed in this substudy, instructs the person to indicate participation in any of the ten roles in the past (up until one week ago), present (today and the preceding week), and anticipated future (tomorrow and any time after tomorrow). The participant's responses to the part one of the Role Checklist are applied to determine the person's participation (or nonparticipation) in each of the listed roles in the past, present, and anticipated future. Scores pertaining to present role participation can also be summed, such that higher sum scores indicate participation in more roles. The roles assessed with the Role Checklist has been found to be consistent with the ICF framework [4], to be linked with the MOHO levels of both performance and participation [3], and to demonstrate concurrent validity with the more extensive Occupational Circumstances Assessment Interview and Rating Scale [5].

Previous translations of the Role Checklist into French [23] and Brazilian Portuguese [24] have both demonstrated good validity and reliability when used in their respective cultures. In addition, measurement properties have been shown to be equivalent when used in paper and electronic version [25], and later modifications of the checklist have similarly been tested with good results [26]. For this study, two independent Norwegian translations of the original instrument were produced and subsequently compared with one another. The comparisons are aimed at ensuring the clarity of concepts as well as the appropriate Norwegian phrasing and sentence structure. After reaching consensus on the forward translation, the instrument was translated back into English, and the back-translated version was checked against the original. Only small modifications were made after this process. Formal guidelines for the translation and adaptation of the Role Checklist have been provided at a later stage in the research process [27]. In addition to completing part one of the Role Checklist, the participants were asked about their age (in years) and gender.

### 2.3. Data Analysis

Descriptive statistics were used to examine the sample characteristics and their participation in roles. Differences between age groups with a view to the proportions currently participating in each role were examined by the *χ*^2^ test. Differences between age groups with regard to the number of currently enacted roles were examined with one-way analysis of variance (ANOVA). In the ANOVA pairwise comparisons, Tukey's honest significant difference was used to adjust for inflating error levels.

Role patterns were described according to Hachey and coworkers [15]. Role loss was defined as having a role in the past, but not in the present. Role gain was defined as having a role at present, but not in the past. Continuous role was defined as having a role in the past, present, and expected future. Role absence was defined as not having the role in the past, not at present, and not in the expected future. By extending Hachey and coworkers' [15] procedure, expected role loss was defined as having a role at present, while expecting not to have the role in the future. Expected role gain was defined as not having a role at present, while expecting to have the role in the future. All analyses were performed with the software IBM SPSS for Windows [28], and the statistical significance was set at *p* < 0.05.

### 2.4. Ethics

All participants were verbally informed about the study by the recruitment collaborator. Written information about the study procedures was provided on the front page of the employed checklist. All participants volunteered to take part in the study, and informed consent was implied when we received the completed forms. As neither health-related information nor any person-identifying information was collected for the study, formal ethical approval was not required.

## 3. Results

### 3.1. Participants

One hundred and forty persons, 49 (35%) men and 91 (65%) women, opted to participate in the study. The age distribution showed that 45 (32.1%) of the participants were aged 18–24 years, 47 (33.6%) were aged 25–44 years, 31 (22.1%) were aged 45–64 years, and 17 (12.1%) were aged 65 years or older. The sample mean age was 40.2 years (SD = 18.8 years). The gender proportions did not differ significantly between the four age groups (*χ*^2^ = 1.26, *p* = 0.74).

### 3.2. Role Participation

**Figure 1:**
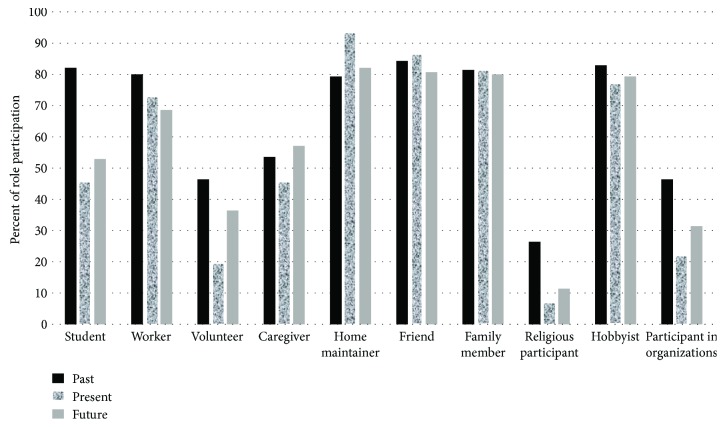
Role participation for past, present, and anticipated future roles in the sample (*n* = 140).

Role participation in the past, present, and anticipated future is displayed in Figure 1. In the total sample, the most frequently endorsed role was home maintainer at present (93.6%), while the least frequently endorsed role was religious participant at present (7.1%). In the youngest age group (18–24 years), the most frequently endorsed role was home maintainer at present (*n* = 45, 100% within the age group), while the least frequently endorsed role was religious participant at present (*n* = 3, 6.7%). In the adult age group (25–44 years), the most frequently endorsed role was home maintainer at present (*n* = 46, 97.9%), while the least frequently endorsed role was religious participant at present (*n* = 0, 0%). Among the middle-aged participants (45–64 years), the most frequently endorsed role was home maintainer at present (*n* = 31, 100%), while the least frequently endorsed role was student at present (*n* = 4, 12.9%). Among the oldest participants (65 years and over), the most frequently endorsed roles were worker in the past and family member at present (both *n* = 31, 100%), while the least frequently endorsed roles were student at present (*n* = 1, 5.9%), student in the future (*n* = 1, 5.9%), and worker in the future (*n* = 1, 5.9%).

### 3.3. Current Role Participation according to Age Groups

Table 1 displays the age group comparisons related to currently enacted roles. Statistically significant differences between the age groups were found for the following roles: student, worker, volunteer, caregiver, home maintainer, friend, and religious participant. In addition, the mean number of currently enacted roles was different between participants in the four age groups. The pairwise comparisons revealed that those in the oldest age group participated in fewer roles compared to participants in all other age groups (all *p* < 0.001). Otherwise, no significant differences occurred.

### 3.4. Continuity and Change in Roles

Table 2 displays the participants' role patterns—that is, the continuity and change in roles across time—as interpreted from the participants' combinations of self-reported past, present, and anticipated future role participation. The roles most frequently lost were student and volunteer, whereas the roles most frequently gained were worker and home maintainer. Home maintainer and friend were the most frequent continuous roles, whereas religious participant and participant in organizations were the roles that most often showed an absent pattern. The roles most frequently expected to be lost were worker and home maintainer. Conversely, the roles most frequently expected to be gained were volunteer and caregiver. Of the predefined role patterns, most of the demonstrated patterns were examples of continuous roles (38.4%).

Considering that the participants in the oldest age group had significantly fewer roles compared to their younger counterparts (see Table 1), there was reason also to examine whether their role patterns across time deviated from the patterns shown for the total sample. The results from this additional analysis of continuity and change in roles for the participants in the oldest age group are displayed in Table 3. In comparison with the total sample, participants in the oldest age group had a higher proportion of the detected role patterns related to role loss and a smaller proportion of the role patterns related to continuous roles and expected role gains.

## 4. Discussion

The current study is aimed at examining participation in roles and patterns of role participation across time, in a general population sample from Norway. Several of the roles listed on the Role Checklist were currently held among a majority of the sample participants. In the high end of the scale, more than 90% of the sample participated in the role home maintainer. In the low end, fewer than 10% of the sample participated in the role religious participant. Participants over the age of 65 had fewer roles compared to their younger counterparts and showed a somewhat different pattern of continuity and change in role participation across time.

The roles listed in the Role Checklist [16] comprise different levels of necessity—some can be viewed as an integral and inescapable aspect of independent living, such as the most frequently endorsed roles of worker and in particular home maintainer (see Figure 1). Other roles, such as the hobbyist and religious participant roles, are not connected to independent living. It is fully possible to lead both independent and fulfilling lives without these roles, and therefore, adopting these roles needs other sources of motivation. Empirical studies have shown that differently composed samples will have each own unique composition of the roles in which participants engage more and less frequently (compare, e.g., [7, 9, 12, 20, 29]). On the other hand, one should be able to assume that role participation in general has a relationship to basic human needs. As suggested by Deci and Ryan [30], such “human motivational universals” are comprised from basic needs for mastery, affiliation, and autonomy. In line with such a view, one may see the worker and home maintainer roles as linked with the need for autonomy, while roles like family member and religious participant link more strongly with the need for affiliation. However, affiliation needs may be expressed and met in different ways. People who live without a family may, for example, rely more on friends, neighbors, and interest groups as sources of companionship and support. This line of reasoning mirrors the recently introduced Model of Occupational Wholeness [31–33], suggesting that personal satisfaction with what we do is based on our tacit judgment of how our doing contributes to our satisfaction of needs. Taken together, this suggests that a majority of participants in any given sample will be expected to participate in some roles (worker, home maintainer, and friend), whereas the level of participation in other roles (e.g., hobbyist, religious participant, and participant in organizations) will depend more on the specific sample characteristics and the given cultural and environmental context [1, 34].

In line with the theoretical assumptions [1, 13, 17, 18], participants in the age group of 65 years and over participated in fewer roles than their counterparts of younger age (see Table 1). The participants in the oldest age group also experienced role loss more frequently compared to the younger participants (see Tables 2 and 3). Reduced role participation may be associated with reduced physical capacity and the onset of illness and functional limitations, which is more often the case in older years. In addition, reaching the normal retirement age (which in Norway is 67 years) directly implies loss of the worker role. Indirectly, it opens up the possibility that friends and family members of similar age may fall ill and eventually die. Consequently, roles like friend, family member, and caregiver may also be lost in the natural progression through life. Nonetheless, occupational therapists should be aware of the potential threats related to role loss. According to the MOHO [1], people organize their everyday lives according to roles. We do what we do much because we enact our roles as researchers, rock band guitarists, amateur kitchen chefs, and a multitude of others. Losing roles as an organizing principle for our everyday lives may further lead to a loss of meaning and purpose.

In most cases, people value the roles they have. In a previous Norwegian study, occupational therapy students demonstrated positive and statistically significant associations between currently enacted roles and their valuation of the same roles [7]. Further, participating in more valued roles appears to lead to more satisfaction, compared to having fewer roles. In support of this, researchers have demonstrated significant associations between participation in more valued roles and having higher quality of life [9] and more life satisfaction [35]. Thus, occupational therapists should pay attention to role loss. Such loss may indicate that the client may be at risk of experiencing reduced quality of life, and he or she may need help to remediate his or her role composition to restore life satisfaction.

In a study conducted in the midnineties, Dickerson and Oakley [12] compared an American nondisabled community sample with a matched sample of persons with physical or mental disabilities. They found that participants in the disabled sample not only had significantly fewer roles compared with the nondisabled sample but also had anticipated fewer future roles and tended to place lower value on several roles, compared to their nondisabled counterparts. The present study augments these previous results by indicating that role loss may not only be an effect of illness and disability but can also be an effect of the aging process itself, regardless of illness or disability (see Tables 2 and 3). At the same time, however, participants in the oldest age group did experience substantial role continuity across the past, present, and anticipated future. Moreover, building on Hachey and coworkers' [15] concept of “role change,” we found that the possibility of *gaining* a role in the future was in fact expected by some of the older participants (Table 3). Two participants expected to gain the role of volunteer, one expected to become a caregiver, and one expected to enter the hobbyist role. Thus, in spite of reaching older age, role participation does not have to be all about losing roles, although this should also be expected.

### 4.1. Study Strengths and Limitations

The sample size, in particular for the oldest age group, was small. Thus, the reliability of the results for this age group is uncertain—there may not have been sufficient variability within the group, and this may have caused biased results. Further studies using larger sample subgroups are needed to shed light on the potential age differences in role participation. Participants were also recruited by convenience, such that we are unable to speak about how well they represent the Norwegian general population. The cross-sectional study design precludes us from establishing cause-effect relationships between the study variables. A trusted group of students and occupational therapist colleagues were used as collaborators in recruiting the participants and informing them about the study procedures. In addition, the employed Role Checklist is relatively straightforward to use, so there is good reason to trust the validity of the collected data. It provides, however, one particular set of roles with a particular set of definitions. Other ways of conceptualizing and measuring role participation may lead to different results. Thus, a limitation of the study is the use of a single measure to look at a complex phenomenon. Moreover, a weakness is the focus on role participation only, not taking into account the participants' valuation of the roles. In addition, potentially associated outcomes, like measures of health or quality of life, were not included. Thus, although previously demonstrated in a clinical sample [9], the study renders the question of whether role participation is related to significant outcomes in a general population sample. If indeed related to such outcomes, we should also ask whether role participation is important in and of itself or whether role participation only matters when the roles in question are personally valued. Future research may also include the Role Checklist in a variety of longitudinal rehabilitation studies to examine whether role participation outcomes are affected as a result of intervention, be they interprofessional or occupational therapy specific.

## 5. Conclusion

The most frequently endorsed current role in the sample was home maintainer, while the least frequently endorsed current role was religious participant. Compared with the total sample, the participants in the oldest age group participated less frequently in most listed roles, and they had a lower total number of current roles. The role patterns for the total sample indicated for the most part a continuity of roles, while the most frequent role pattern for the oldest group of participants was role loss. More health-related problems, but also the reaching retirement age itself, may lead to role loss as one moves towards older age. Rehabilitation services in general and participation-focused occupational therapy in particular may profit from assessing role participation in clients and potentially target roles through intervention.

## Figures and Tables

**Table 1:** Frequency and proportion of individuals currently participating in roles according to age groups.

	Age groups				
Current roles	18–24 years *n* (%)	25–44 years *n* (%)	45–64 years *n* (%)	≥65 years *n* (%)	*p*
Student	43 (95.6)	16 (34.0)	4 (12.9)	1 (5.9)	<0.001
Worker	31 (68.9)	38 (80.9)	30 (96.8)	3 (17.6)	<0.001
Volunteer	8 (17.8)	8 (17.0)	11 (35.5)	0 (0.0)	0.02
Caregiver	9 (20.0)	30 (63.8)	19 (61.3)	6 (35.3)	<0.001
Home maintainer	43 (95.6)	46 (97.9)	31 (100.0)	11 (64.7)	<0.001
Friend	45 (100.0)	41 (87.2)	23 (74.2)	12 (70.6)	<0.01
Family member	33 (73.3)	39 (83.0)	26 (83.9)	18 (94.1)	0.27
Religious participant	3 (6.7)	0 (0.0)	5 (16.1)	2 (11.8)	<0.05
Hobbyist	34 (75.6)	37 (78.8)	24 (77.4)	13 (76.5)	0.99
Participant in organizations	7 (15.6)	11 (23.4)	9 (29.0)	4 (23.5)	0.56
	*M* (SD)	*M* (SD)	*M* (SD)	*M* (SD)	
Number of current roles	5.69 (1.56)	5.66 (1.20)	5.87 (1.65)	4.0 (1.37)	<0.001

*Note*. Differences by *χ*^2^ tests for separate roles and by one-way ANOVA *F*-test for the number of current roles.

**Table 2:** Continuity and change in role participation across time in the total sample (*n* = 140).

Role	Loss	Gain	Continuous	Absence	Expected loss	Expected gain
	*n* (%)	*n* (%)	*n* (%)	*n* (%)	*n* (%)	*n* (%)
Student	61 (43.6)	10 (7.1)	49 (35.0)	8 (5.7)	11 (7.9)	21 (15.0)
Worker	34 (24.3)	24 (17.1)	75 (53.6)	2 (1.4)	23 (16.4)	17 (12.1)
Volunteer	48 (34.3)	10 (7.1)	15 (10.7)	51 (36.4)	10 (7.1)	34 (24.3)
Caregiver	24 (17.1)	13 (9.3)	50 (35.7)	34 (24.3)	8 (5.7)	24 (17.1)
Home maintainer	3 (2.1)	23 (16.4)	107 (76.4)	3 (2.1)	19 (13.6)	3 (2.1)
Friend	15 (10.7)	18 (12.9)	103 (73.6)	3 (2.1)	17 (12.1)	9 (6.4)
Family member	18 (12.9)	18 (12.9)	94 (67.1)	4 (2.9)	18 (12.9)	16 (11.4)
Religious participant	27 (19.3)	0 (0.0)	10 (7.1)	100 (71.4)	0 (0.0)	6 (4.3)
Hobbyist	22 (15.7)	14 (10.0)	92 (65.7)	7 (5.0)	14 (10.0)	17 (12.1)
Participant in organizations	40 (28.6)	6 (4.3)	24 (17.1)	63 (45.0)	6 (4.3)	19 (13.6)
Total	292	136	619	275	126	166
Role patterns	18.1%	8.4%	38.4%	17.0%	7.8%	10.3%

*Note*. Role loss was defined as having a role in the past, but not in the present. Role gain was defined as having a role at present, but not in the past. Continuous role was defined as having a role in the past, present, and expected future. Role absence was defined as not having the role in the past, not at present, and not in the expected future. Expected role loss was defined as having a role at present, while expecting not to have the role in the future. Expected role gain was defined as not having a role at present, while expecting to have the role in the future.

**Table 3:** Continuity and change in role participation across time among the participants in the oldest age group (*n* = 17).

Role	Loss	Gain	Continuous	Absence	Expected loss	Expected gain
	*n* (%)	*n* (%)	*n* (%)	*n* (%)	*n* (%)	*n* (%)
Student	14 (82.4)	0 (0.0)	1 (5.9)	2 (11.8)	0 (0.0)	0 (0.0)
Worker	14 (82.4)	1 (5.9)	1 (5.9)	0 (0.0)	2 (11.8)	0 (0.0)
Volunteer	11 (64.7)	0 (0.0)	0 (0.0)	5 (29.4)	0 (0.0)	2 (11.8)
Caregiver	9 (52.9)	2 (11.8)	4 (23.5)	2 (11.8)	0 (0.0)	1 (5.9)
Home maintainer	3 (17.6)	3 (17.6)	7 (41.2)	3 (17.6)	3 (17.7)	0 (0.0)
Friend	3 (17.6)	2 (11.8)	10 (58.8)	2 (11.8)	2 (11.8)	0 (0.0)
Family member	1 (5.9)	3 (17.6)	13 (76.5)	0 (0.0)	2 (11.8)	0 (0.0)
Religious participant	3 (17.6)	0 (0.0)	2 (11.8)	12 (70.6)	0 (0.0)	0 (0.0)
Hobbyist	4 (23.5)	4 (23.5)	8 (47.1)	0 (0.0)	4 (23.5)	1 (5.9)
Participant in organizations	8 (47.1)	0 (0.0)	4 (23.5)	5 (29.4)	0 (0.0)	0 (0.0)
Total	70	15	50	31	13	4
Role patterns	38.3%	8.2%	27.3%	16.9%	7.1%	2.2%

*Note*. Role loss was defined as having a role in the past, but not in the present. Role gain was defined as having a role at present, but not in the past. Continuous role was defined as having a role in the past, present, and expected future. Role absence was defined as not having the role in the past, not at present, and not in the expected future. Expected role loss was defined as having a role at present, while expecting not to have the role in the future. Expected role gain was defined as not having a role at present, while expecting to have the role in the future.

## Data Availability

The data by which the conclusions of this study is built can be accessed from the author upon reasonable request.
